# Root causes and outcomes of postoperative pulmonary complications after abdominal surgery: a retrospective observational cohort study

**DOI:** 10.1186/s13037-019-0221-5

**Published:** 2019-12-03

**Authors:** Antero Fernandes, Jéssica Rodrigues, Patrícia Lages, Sara Lança, Paula Mendes, Luís Antunes, Carla Salomé Santos, Clara Castro, Rafael S. Costa, Carlos Silva Lopes, Paulo Matos da Costa, Lúcio Lara Santos

**Affiliations:** 1 Experimental Pathology and Therapeutics Group, Instituto Português de Oncologia, Porto, Portugal; 2 Polyvalent Intensive Care Unit of Intensive Medicine Service, Hospital Garcia de Orta, E.P.E, Almada, Portugal; 3 Cancer Epidemiology Group, IPO Porto Research Center (CI-IPOP), Instituto Português de Oncologia, Porto, Portugal; 4 General Surgery Service, Hospital Garcia de Orta, E.P.E, Portugal and Faculdade de Medicina da Universidade de Lisboa, Almada, Portugal; 5 Polyvalent Intensive Care Unit, Hospital Santo Espírito ilha Terceira, E.P.R, Angra do Heroísmo, Açores Portugal; 6 Surgical Oncology Department of Portuguese Instituto Português de Oncologia, Porto, Portugal; 7 EPIUnit - Institute of Public Health, Universidade do Porto, Porto, Portugal; 8 IDMEC, Instituto Superior Técnico, Universidade de Lisboa, Lisbon, Portugal; 9 REQUIMTE/LAQV, Department of Chemistry, Faculty of Science and Technology, Universidade Nova de Lisboa, Caparica, Portugal; 10 Biomedical Sciences Institute Abel Salazar, Universidade do Porto, Porto, Portugal

**Keywords:** Abdominal surgery, Acute respiratory failure, Mechanical ventilation, Polyvalent intensive care unit, Postoperative pulmonary complications, Risk score

## Abstract

**Background:**

Postoperative pulmonary complications (PPCs) contribute significantly to overall postoperative morbidity and mortality. In abdominal surgery, PPCs remain frequent. The study aimed to analyze the profile and outcomes of PPCs in patients submitted to abdominal surgery and admitted in a Portuguese polyvalent intensive care unit.

**Methods:**

From January to December 2017 in the polyvalent intensive care unit of Hospital Garcia de Orta, Almada, Portugal, we conducted a retrospective, observational study of inpatients submitted to urgent or elective abdominal surgery who had severe PPCs. We evaluated the perioperative risk factors and associated mortality. Logistic regression was performed to find which perioperative risk factors were most important in the occurrence of PPCs.

**Results:**

Sixty patients (75% male) with a median age of 64.5 [47–81] years who were submitted to urgent or elective abdominal surgery were included in the analysis. Thirty-six patients (60%) developed PPCs within 48 h and twenty-four developed PPCs after 48 h. Pneumonia was the most frequent PPC in this sample. In this cohort, 48 patients developed acute respiratory failure and needed mechanical ventilation. In the emergency setting, peritonitis had the highest rate of PPCs. Electively operated patients who developed PPCs were mostly carriers of digestive malignancies. Thirty-day mortality was 21.7%. The risk of PPCs development in the first 48 h was related to the need for neuromuscular blocking drugs several times during surgery and preoperative abnormal arterial blood gases. Median abdominal surgical incision, long surgery duration, and high body mass index were associated with PPCs that occurred more than 48 h after surgery. The American Society of Anesthesiologists physical status score 4 and COPD/Asthma determined less mechanical ventilation needs since they were preoperatively optimized. Malnutrition (low albumin) before surgery was associated with 30-day mortality.

**Conclusion:**

PPCs after abdominal surgery are still a major problem since they have profound effects on outcomes. Our results suggest that programs before surgery, involve preoperative lifestyle changes, such as nutritional supplementation, exercise, stress reduction, and smoking cessation, were an effective strategy in mitigating postoperative complications by decreasing mortality.

## Background

Nearly 234 million patients undergo major surgery worldwide every year [1]. Approximately 16% will suffer a complication within 30 days [2]. In 2015, the European Perioperative Clinical Outcome (EPCO) standardized the concept of postoperative complications (POCs) in the various organ systems, which constitutes an important advance in perioperative medicine [3]. One set of under-reported complications are postoperative pulmonary complications (PPCs) that are costly and increase patient mortality. After abdominal surgery (AS), PPCs are one of the most important causes of postoperative morbidity and mortality [4, 5]. Various perioperative risk factors are related to their appearance in the postoperative period [6]. Physiological changes in the respiratory system that occur immediately after the induction of general anesthesia explain the majority of POCs [7]. Thus, respiratory drive and muscle function are altered, lung volumes reduced, and atelectasis develops in more than 75% of patients receiving neuromuscular blocking drugs (NMBD). The respiratory system may take 6 weeks to return to its preoperative state after general anesthesia for major surgery [8]. Acute respiratory failure (ARF) is common in intensive care settings and classified by some studies as a PPC on its own, and in some patients its severity that may lead to the need for mechanical ventilation (MV) as a method of respiratory support [9]. Previous respiratory pathology, obesity, and nutritional deficits also contribute significantly to the occurrence of respiratory complications in the postoperative period. Some of these factors are modifiable [10, 11]. Facing the magnitude of PPCs, performing an early identification of surgical patients at risk for ARF would allow to intervene in an earlier and most useful time, increasing the survival of these patients [12–17]. The American Society of Anesthesiologists physical status (ASA PS) and ARISCAT score (for PPCs), while effective risk prediction tools, can help reduce morbidity and mortality [18, 19]. However, there are few prospective comparative studies of accuracy between them, and this knowledge is still poorly investigated [20, 21]. The rationale of this study was to analyze the incidence, precocity, profile and the outcome impact of PPCs in patients who underwent AS and were admitted in a PICU (polyvalent intensive care unit) in order to find strategies that minimize their mortality.

## Methods

Study patients were included from a universe of medical and surgical patients hospitalized in PICU of Hospital Garcia de Orta, Almada, Portugal, from January to December 2017. Sixty patients who were submitted to an urgent or elective AS and developed PPCs in the postoperative period leading to severe ARF despite the need for MV were retrospectively analyzed. The admission criteria in PICU were the surgical complexity level 4 and 5 according to L. R. Pasternak classification (meaning highly invasive procedure, duration of surgery and intraoperative complications with usual postoperative PICU stay with invasive monitoring) and severity criteria (Table 1) of the patient [22]. All patients received standard clinical care and no research-related intervention was introduced. An experienced chest physician assessed the postoperative respiratory status of all patients. We collected data on the occurrence (≤48 h and > 48 h) of symptomatic and clinically significant PPCs using clinical, laboratory, and radiology data. We evaluated perioperative risk factors associated with PPCs, namely age and gender, body mass index (BMI), previous history of chronic obstructive pulmonary disease (COPD), serum albumin, type of anesthesia (general versus spinal anesthesia), type of surgery (laparotomy versus laparoscopy), use of NMBD during perioperative period, incision type, surgical intervention time and surgical procedure in urgent or elective context. PPCs have been defined according to EPCO and were diagnosed by clinical and radiological examinations and arterial blood gases (ABG), examined using the ABL 555 analyzer (Radiometer, Copenhagen, Denmark). Using clinical records, the risk of PPC was retrospectively estimated according to ASA PS and ARISCAT scores, and this data was compared with the real incidence. Right after surgery and 48 h after AS, pulmonary examinations of patients were repeated and when PPCs were present the following classifications were registered:

**Table 1:** Severity criteria on admission, PICU Length of stay and mortality of patients undergoing AS

Admitted patients	60 cases
PICU hospitalization time in days (median, min-max) APACHE II (mean ± standard deviation) SAPS II (mean ± standard deviation)	6.31 days [0.8–21] 22.8 ± 8.1points 50.7 ± 17.9points
PICU Mortality rate (n, %)	21.7% (13 patients)
Hospital mortality rate (n, %)	36.7% (22 patients)
SMR for SAPS II (median, min-max)	0.68 points [0.53–0.74]
Readmission rate < 48 h (%)	0.9%
VAP, number of episodes/1000 days of IT (median, min-max)	8.7 [7.1–10.3]

*APACHE* Acute Physiology, Age, Chronic Health Evaluation, *AS* abdominal surgery, *IT* tracheal intubation, *PICU* polyvalent intensive care unit, *PPCs* Postoperative pulmonary complications, *SAPS* Simplified Acute Physiology Score, *SMR* Standardized Mortality Rate, *VAP* ventilator-associated pneumonia

1. Atelectasis (by thoracic ultrasound, CT scan and/or x-ray evidence of the collapse of the alveoli, lung opacification with the shift of the mediastinum, hilum, or hemidiaphragm toward the affected area, and compensatory over inflation in the adjacent non-atelectatic lung);

2. Bronchospasm (newly detected expiratory wheezing treated with bronchodilators), pleural effusion (chest radiograph demonstrating; blunting of the costophrenic angle, evidence of displacement of adjacent anatomical structures, or (in supine position) a hazy opacity in one hemithorax with preserved vascular shadows);

3. Pneumothorax (a collection of air in the pleural space - an area with no vascular bed surrounding the visceral pleura);

4. Acute Respiratory Distress Syndrome (ARDS) diagnosed by criteria of Berlin definition 2012 [23];

5. Pulmonary emboli (diagnosed if a patient had suggestive clinical findings, blood gas abnormality, consistent image of a pulmonary embolism on computed tomography with intravenous contrast);

6. Pneumonia (diagnosed if a patient had clinical, laboratory and/or radiological evidence of consolidation or infiltration not present in the preoperative chest roentgenograms), with or without positive cultures;

7. Tracheobronchitis (diagnosed if the patient had clinical, laboratory and no radiological evidence of consolidation or infiltration in chest roentgenograms);

8. Aspiration pneumonitis (acute lung injury after the inhalation of regurgitated gastric contents);

9. ARF (postoperative arterial oxygen pressure (PaO2) < 60 mmHg on room air, a ratio of PaO2 to inspired oxygen fraction (FiO2) < 300, or arterial oxygen saturation (SaO2) < 90% and requiring oxygen therapy).

10. Patient optimization means control of chronic diseases and kinesiotherapy.

11. The term “prehabilitation”, is a combination of the words “pre- “and “rehabilitation”. Prehabilitation concerns a combination of preparational and post-procedure measures to improve the outcome of a planned procedure, such as major surgery. Prehabilitation programs are used to improve postoperative outcomes. These programs before surgery, involve preoperative lifestyle changes, such as nutritional supplementation, exercise, stress reduction, and smoking cessation. Therefore structured and sustained exercise over a period of few weeks leads to improved cardiovascular, respiratory, and muscular conditioning.

The incidence of PPCs, the profile and the postoperative mortality associated defined as death within 30 days of surgery were also evaluated.

All statistical analyses were performed with R Statistical software (version 3.6.0). Continuous variables were described using median and range or mean ± standard deviation, and categorical variables were expressed as frequencies or percentages. Student’s t-tests were used for comparing continuous variables, and chi-squared tests or Fisher’s exact tests were used for comparing categorical data. A *p*-value of < 0.05 was considered to be statistically significant. Logistic regression was performed to determine which perioperative risk factors were associated with the development of PPCs.

The Ethics Committee of Hospital Garcia de Orta approved the study protocol.

## Results

In our sample, 45 (75%) males and 15 (25%) females, with a median (min-max) age of 64.5 years (47–81) were submitted to urgent or elective abdominal surgery. Severity criteria on admission, PICU Length of stay and mortality of patients undergoing AS are shown in Table 1. Thirty six patients underwent emergency surgery and the remaining 24, elective surgery (Table 2).

**Table 2:** AS etiology in patients with PPCs

Etiology	Patients (n, %)	Emergency surgery (n, %)		Elective surgery (n, %)	
		≤48 h	> 48 h	≤48 h	> 48 h
Peritonitis	11 (18.3)	9 (15.0)	2 (3.3)	–	–
Colorectal cancer	9 (15.0)	–	2 (3.3)	7 (11.7)	–
Mesenteric ischemia	8 (13.3)	1 (1.7)	7 (11.7)	–	–
Cholangiocarcinoma	5 (8.3)	–	–	3 (5.0)	2 (3.3)
Bowel obstruction	4 (6.7)	3 (5.0)	1 (1.7)	–	–
Abdominal trauma	3 (5.0)	2 (3.3)	1 (1.7)	–	–
Acute Pancreatitis	3 (5.0)	–	3 (5.0)	–	–
Gastric cancer	5 (8.3)	–	–	4 (6.7)	1 (1.7)
Esophageal cancer	5 (8.3)	–	–	5 (8.3)	–
Cholecystitis	5 (8.3)	2 (3.3)	3 (5.0)	–	–
Diverticulitis	1 (1.7)	–	–	–	1 (1.7)
Abdominal aortic aneurysm	1 (1.7)	–	–	–	1 (1.7)

### PPC group description

Thirty-six patients (60%) developed PPCs within 48 h, from which 29 (80.6%) were male. The median (min-max) age was 65 (54–81) years. Twelve patients (20%) developed additional PPCs related with invasive ventilation side effects after 48 h. Twenty-four (40%) only developed PPCs after 48 h. From these, 16 (66.7%) were male. The median (min-max) age was 64.5 (49–81) years. We did not find significant differences in the median age of the two groups (*p* = 0.2).

**Fig. 1:**
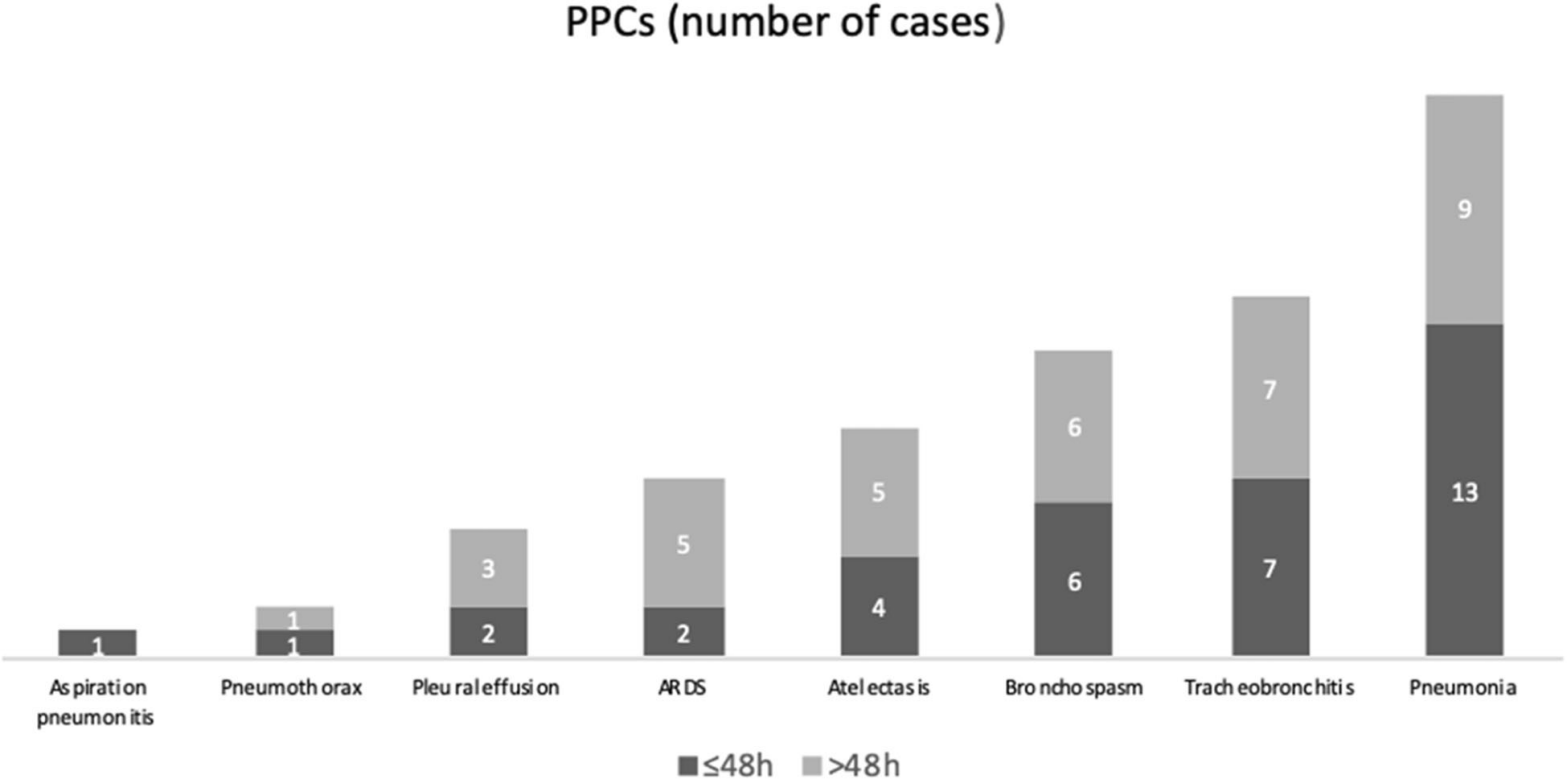
Postoperative pulmonary complication in the first 48 h and after 48 h

Pneumonia was the most frequent PPC in the sample (PPCs ≤48 h and > 48 h; Fig. 1). Regarding the patients who developed additional PPCs after 48 h, the complications were 7 tracheobronchitis, 6 bronchospasms, 5 atelectasis, 5 ARDS, 3 pleural effusion and 1 pneumothorax.

In the emergency setting, peritonitis had the highest rate of PPCs. The group of electively treated patients who developed PPCs was mostly carriers of digestive malignancies (Table 2).

### Perioperative relevant risk factor for the development of PPCs

Regarding the preoperative variables and the risk of PPCs, we verified that the knowledge of ASA score 4 before surgery associated with measures to optimize performance status revealed to be a PPC protection factor (OR = 0.04; 95%CI: 0.01–0.28). Patients with a high risk ARISCAT score also showed a reduced risk of developing PPCs after 48 h (OR = 0.17; 95%CI: 0.03–0.88). In this group of patients, preoperative performance status optimization and/or early diagnosis of pulmonary impairment reflected a reduced risk of PCCs, as we mentioned before. A significantly lower risk of PCCs was also observed for patients undergoing a median surgical incision, in the first 48 h (OR = 0.22; 95%CI: 0.06–0.79), and for patients with more time-consuming surgeries, after 48 h (OR = 0.29; 95%CI: 0.09–0.90). In contrast, patients with pre-operative abnormal arterial blood gases prior to surgery (OR = 3.50; 95%CI: 1.14–10.74) or who required NMBD several times intraoperatively had a higher risk of PPCs in the first 48 h (OR = 18.40; 95%CI: 2.24–151.35). High BMI was significantly associated with the occurrence of PPCs after 48 h (OR = 15.40; 95%CI: 1.47–160.97) (Table 3).

**Table 3:** Potential perioperative risk factors and PPCS, Mechanical Ventilation and 30-day mortality

Variable	n	PPC (Postoperative Pulmonary Complications)		Mechanical Ventilation	30-Day Mortality
		≤ 48 h	> 48 h		
		OR (95% C.I.)^a^	OR (95% C.I.)^a^	OR (95% C.I.)^a^	OR (95% C.I.)^a^
Sex					
M	45	1	1	1	1
F	15	0.48 (0.15–1.58)	3.50 (0.87–14.11)	0.59 (0.15–2.35)	2.31 (0.62–8.64)
NMBD					
No	43	1	1	1	1
Yes	17	**18.40 (2.24–151.35)**	0.67 (0.21–2.08)	**	2.81 (0.78–10.12)
Age	60	1.05 (0.98–1.13)	0.95 (0.89–1.03)	1.01 (0.93–1.10)	1.05 (0.97–1.15)
ASA					
1	18	1	1	1	1
2	17	0.37 (0.07–1.80)	0.56 (0.14–2.21)	0.94 (0.05–16.35)	1.09 (0.27–4.41)
3	13	0.32 (0.06–1.70)	0.31 (0.07–1.38)	0.13 (0.01–1.37)	0.17 (0.02–1.60)
4	12	**0.04 (0.01–0.28)**	2.50 (0.41–15.23)	**0.06 (0.01–0.59)**	**
ARISCAT					
Low risk < 26	14	1	1	1	1
Intermediate Risk 26–44	16	1.65 (0.37–7.37)	0.21 (0.04–1.29)	0.72 (0.10–5.09)	1.50 (0.32–6.99)
High risk > 45	30	0.98 (0.27–3.53)	**0.17 (0.03–0.88)**	0.55 (0.10–3.06)	0.28 (0.05–1.47)
Respiratory Infection					
No	50	1	1	1	1
Yes	10	1.00 (0.25–4.00)	1.69 (0.39–7.31)	2.54 (0.29–22.27)	1.71 (0.38–7.84)
Anemia					
No	30	1	1	1	1
Yes	30	0.76 (0.27–2.13)	2.33 (0.81–6.73)	1.00 (0.28–3.54)	1.22 (0.36–4.17)
Incision Type					
Bilateral subcostal	21	1	1	1	1
Median	39	**0.22 (0.06–0.79)**	**4.14 (1.34–12.72)**	0.13 (0.02–1.07)	0.55 (0.16–1.91)
LPT vs. LPC					
LPC (Laparoscopy)	19	1	1	1	1
LPT (Laparotomy)	41	1.14 (0.38–3.43)	1.14 (0.38–3.43)	1.73 (0.47–6.40)	0.45 (0.13–1.58)
Surgery Duration					
< 180 min	42	1	1	1	1
> 180 min	18	3.18 (0.90–11.28)	**0.29 (0.09–0.90)**	2.50 (0.49–12.79)	2.50 (0.70–8.92)
Smokers					
No	35	1	1	1	1
Yes	25	1.33 (0.46–3.83)	0.75 (0.26–2.14)	1.00 (0.28–3.61)	0.55 (0.15–2.04)
BMI					
< 18	12	1	1	1	1
18–25	20	0.78 (0.15–3.93)	1.40 (0.33–5.93)	**	0.75 (0.14–4.13)
25–30	16	0.56 (0.11–2.90)	2.33 (0.51–10.78)	**	1.36 (0.25–7.32)
30–35	12	**0.11 (0.02–0.71)**	**15.40 (1.47–160.97)**	**	0.27 (0.02–3.09)
COPD/Asthma History					
No	42	1	1	1	1
Yes	18	0.56 (0.18–1.70)	2.15 (0.65–7.11)	**0.21 (0.06–0.80)**	1.63 (0.45–5.92)
POSA					
≤ 35 g/L	8	5.55 (0.64–48.41)	0.63 (0.14–2.79)	1.88 (0.21–16.92)	**19.29 (3.23–115.22)**
> 35 g/l	52	1	1	1	1
Lactate					
< 4 mmol/l	14	1	1	1	1
> 4 mmol/l	46	0.52 (0.14–1.90)	1.17 (0.35–3.92)	**	0.38 (0.10–1.44)
PREOP Abnormal Arterial blood gases					
No	20	1	1	1	1
Yes	40	**3.50 (1.14–10.74)**	**0.16 (0.04–0.63)**	1.57 (0.43–5.77)	0.49 (0.14–1.74)
Pa02 ≤ 50 ≥ 60 Moderate - Severe ARF					
No	25	1	1	1	1
Yes	35	2.36 (0.82–6.83)	0.41 (0.14–1.23)	1.53 (0.43–5.44)	1.82 (0.49–6.74)
Pa02/FiO2					
< 100	21	0.83 (0.28–2.45)	1.13 (0.38–3.35)	1.80 (0.43–7.53)	**
101–300	39	1	1	1	1
PREOP Abnormal Chest radiography					
No	36	1	1	1	1
Yes	24	3.00 (0.97–9.30)	0.37 (0.13–1.09)	4.23 (0.84–21.40)	2.06 (0.59–7.13)

*ASA PS* American Society of Anesthesiologists physical status, *BMI* body mass index, *COPD* Chronic Obstructive Pulmonary Disease, *FiO*_***2***_ Inspiratory oxygen fraction, *LPC* Laparoscopy, *LPT* Laparotomy, *NMBD* Neuromuscular blocking drugs, *PaO*_*2*_ arterial oxygen pressure, *PREOP* preoperative period, *POSA* preoperative serum albumin. a Unadjusted (Univariable Model);** No cases in at least one of the groups; Bold - significant values.

No significant differences in APACHE and SAPS scores were found between the patients that developed PPCs in the first 48 h and the group of patients that only developed PPCs after 48 h (APACHE: *p* = 0.829; SAPS: *p* = 0.378) (Table 4).

**Table 4:** PPCs, 30-day mortality and Severity Indices

Patients undergoing AS	Patients number (%)	30-day mortality	APACHE II (mean)	SAPS II (mean)
OP	60 (100)	21.7%	22.8 ± 8.1^*^	50.7 ± 17.9^*^
PPCs ≤48 h	36 (60)	27.8% (10 patients)	23.0 ± 6.5^**^	49.0 ± 17.3^**^
PPCs > 48 h	24 (40)	12.5% (3 patients)	22.5 ± 10.2^**^	53.2 ± 18.9^**^

*APACHE* Acute Physiology, Age, Chronic Health Evaluation, *AS* abdominal surgery, *OP* operated patients, *PPCs* postoperative pulmonary complications, *SAPS* Simplified Acute Physiology Score; * (APACHE: *p* = 0.829; SAPS: *p* = 0.378). ** statistically not significant

### Mechanical ventilation and mortality relevant factors

In our sample, 48 patients developed ARF and needed mechanical ventilation, 23 (47.9%) patients within 24 h and 25 (52.1%) after 24 h. In only 1 of these patients, a non-invasive method was the first attempt. Patients with COPD or asthma history and optimized ASA score 4 were less ventilated. All cases with elevated lactate 12 h after surgery developed PPCs and required ventilation. Patients with proven respiratory infection required ventilation within 24 h.

Thirty-day mortality of patients with PPCs was analyzed and its association with the APACHE II and SAPS II scores were assessed. No significant association was found between these scores and 30-day mortality. Mortality was lower in optimized patients (ASA 4: *p* = 0.04). Patients with albumin deficiency before surgery had major and significant mortality in the first 30 days after surgery (*p* = 0.01).

## Discussion

To the best of our knowledge, this is the first study that evaluates the profile of PPCs in a population submitted to abdominal surgery in Portugal. We observed that PPCs occur within 48 h in 60% of abdominal surgical patients that need ICU care in the immediate postoperative period. Patients operated in an emergency setting for peritonitis had the highest rate of PPCs. In the elective setting, patients who were operated due to a digestive cancer were more prone to a PPC. Forty-eight patients developed ARF. In accordance with our results, the report of Serejo et al*.* [24] recorded a 28.2% incidence of pulmonary complications, in patients undergoing emergency abdominal surgery. Kumar et al. studied one hundred and fifty patients who underwent abdominal surgery, and of these, 16% developed PPCs and the highest incidence occurred in the emergency surgery group too [25]. Verma et al. conducted a study of PPCs in patients of emergency abdominal surgeries and found that pre-operative abnormal chest X-ray changes were 3 times more common in the PPCs group as compared to the control group without PPCs [26]. In our study, no significant association was observed between this variable and the occurrence of PPCs. On the other hand, regarding elective surgery, Yang and colleagues [6] confirmed this finding, reporting a higher incidence of PPCs in esophagectomy and other upper abdominal procedures as we found in our series.

We observed that patients with ASA score 4 and high-risk ARISCAT score before surgery had a lower risk of complications within the first 48 h and after 48 h, respectively, as they were previously optimized. Low albumin levels were found to be poor prognostic factor. It is important to underline that in cases of emergency surgery we cannot modify these risk factors. However, in elective surgery, even in digestive oncological diseases, we have time to optimize these patients. In this sense, there is evidence that prehabilitation programs reduced the risk of complications including respiratory ones [27] and such a prehabilitation program is being implemented in our Institutions.

High ARISCAT score, high BMI, pre-operative abnormal arterial blood gases, and albumin deficiency before surgery can be a surrogate marker for prehabilitation measures to improve their prevention. In several studies, the ARISCAT score has proved its efficacy in the identification of PPCs risk in the surgical population, including the population submitted to abdominal surgery. This score was already validated for the Portuguese population [28, 29].

We found that, in the first 48 h after ICU admission, the identification of lactate acidemia or pre-operative abnormal arterial blood gases plays an important role in therapeutic measures with a positive impact on the outcome of these patients. In the group that needed ventilation 24 h after surgery that had alterations in the arterial gasometry and that required neuromuscular blockade several times during surgery had an increased risk of pulmonary complications. Creagh-Brown et al. [30] evaluated the effect of the peak serum lactate, in the first 24 h of ICU admission after major gastrointestinal surgery, in a large cohort of patients from nearly 250 hospitals in the United Kingdom. In that study, they found an increased in-hospital mortality associated with elevated lactate levels, with no difference between elective and emergency surgery. Veličković et al. showed that lactate levels measured at 12 h after the operation had the highest predictive ability for diagnosis of overall postoperative complications including PPCs and the postoperative in-hospital mortality [31]. Therefore, lactates should be monitored in the immediate postoperative because they help to identify the risk of PPCs.

In our series, patients who required NMBD several times intraoperatively developed a higher rate of PPCs in the first 48 h. Recent studies evaluating the use of neuromuscular blocking agents and postoperative complications have demonstrated growing evidence for a clear relationship between the use of these agents and PPCs complications [32].

It is necessary to underline that the need for invasive mechanical ventilation was understood by us as the extreme consequence of a PPC. As a respiratory support technique it is not, by itself, obviously therapeutic, and may be associated with several complications, namely mechanical ventilation lung injury, usually manifested in the form of barotrauma, volutrauma, atelectrauma, biotrauma and more recently ergotrauma, globally inserted in the new energy concept of mechanical power, which is now thought to be the basis of mechanical ventilation lung injury [33, 34]. The severity of mechanical ventilation lung injury is partly dependent on the duration of the injury, which is why the safety and efficacy binomial are two important factors. However, in our series ventilation was not correlated with 30-day mortality.

Fernandez-Bustamante et al. [7] in a recent report of 1202 patients undergoing non-cardiothoracic surgery under general anesthesia, patients with at least one pulmonary complication had higher rates of mortality, ICU admission, and length of stay, and all patients were ASA PS class 3 or greater. In our series 25 patients (41.7%) were classified ASA PS classes 3 and 4, who were responsible for 10 (16.7%) of the PPCs before 48 h and 15 (25%) of the PPCs after 48 h. As mentioned before, patients with ASA score 4 had less PPCs in the first 48 h since they received preoperative optimization.

The 30-days postoperative mortality was higher in PPCs developed within 48 h (27.8%), therefore these complications revealed a high lethality rate. Patel et al. also showed that 30-day mortality was higher in patients undergoing abdominal surgery with PPCs [4]. Patients with albumin deficiency prior to surgery had a higher risk of death in the first 30 days after surgery. Lunardi A et al. showed that malnutrition is associated with weakness of the expiratory muscles, decreased chest wall expansion and increased incidence of pulmonary complications in patients undergoing elective upper abdominal surgery [35].

This study has limitations regarding the size of the sample. On the other hand, the fact that it includes only patients who required intensive care after abdominal surgery makes it more homogeneous. Taking our results together, we consider that it is necessary to define variables that predict lung complications in the postoperative period and to establish strategies for the mitigation of PPCs after surgery.

Duarte and Machado reviewed the epidemiology, risk factors and prevention of PPCs and concluded that the clinical and social consequences of PPCs are huge and that prevention of its high incidence continues to be a growing challenge focusing on the importance of preventive strategies, which should be systematically applied in order to achieve better results [36].

Major AS is a great stressor to patients and causes large physiological changes, leads to tissue trauma, immobility, psychological distress and reduced quality of life [37, 38].

Physical exercise prehabilitation has been proposed to improve postoperative outcomes in patients undergoing major AS. Several studies have been published in the literature investigating the effect of preoperative exercise training compared with standard care on postoperative outcomes in major AS concluding that the effect is beneficial [39].

The improvement of physical capacity through prehabilitation may facilitate better recovery after surgery and the current evidence is that prehabilitation protocols and optimization of preoperative care, in particular, respiratory function, may reduce PPCs incidence and mortality [40–42]. In this sense, it is important to study more comprehensive preoperative risk scores such as P-Possum and ACS NSIQ Risk Calculator to better identify risk patients [43–48].

## Conclusions

PPCs after abdominal surgery are still a major problem since they have profound effects on outcomes. Our results suggest that programs before surgery, involve preoperative lifestyle changes, such as nutritional supplementation, exercise, stress reduction, and smoking cessation, recently defined as prehabilitation, was an effective strategy in mitigating postoperative complications by decreasing mortality.

## Abbreviations

ABG: Arterial Blood Gases
ARDS: Acute Respiratory Distress Syndrome
ARF: Acute Respiratory Failure
ARISCAT: Assess Respiratory Risk in Surgical Patients in Catalonia
AS: Abdominal Surgery
ASA PS: Anesthesiologists Physical Status
BMI: Body Mass Index
COPD: Chronic Obstructive Pulmonary Disease
EPCO: European Perioperative Clinical Outcome
MV: Mechanical Ventilation
NMBD: Neuromuscular Blocking Drugs
PICU: Polyvalent Intensive Care Unit
POCs: Postoperative Complications
PPCs: Postoperative Pulmonary Complications
